# Role of three tick species in the maintenance and transmission of Severe Fever with Thrombocytopenia Syndrome Virus

**DOI:** 10.1371/journal.pntd.0008368

**Published:** 2020-06-10

**Authors:** Yuan-Yuan Hu, Lu Zhuang, Kun Liu, Yi Sun, Ke Dai, Xiao-Ai Zhang, Pan-He Zhang, Zhi-Chun Feng, Hao Li, Wei Liu

**Affiliations:** 1 Graduate School of Anhui Medical University, Hefei, P. R. China; 2 State Key Laboratory of Pathogen and Biosecurity, Beijing Institute of Microbiology and Epidemiology, Beijing, P. R. China; 3 Affiliated Bayi Children’s Hospital, The 7th Medical Center of People’s Liberation Amy General Hospital, Beijing, P. R. China; 4 School of Public Health, Air Force Medical University, Xi’an, Shaanxi, P. R. China; Institute for Disease Modeling, UNITED STATES

## Abstract

Severe fever with thrombocytopenia syndrome virus (SFTSV) is a novel phlebovirus in the Bunyaviridae family, causing SFTS with high mortality rate. *Haemaphysalis longicornis* ticks has been demonstrated as a competent vector of SFTSV by experimental transmission study and field study. However, there has been query whether other tick species that infest human beings in the SFTS endemic regions are capable of transmitting the pathogen. Here by performing experimental transmission study, we compared the capable of transmitting SFTSV among *Ixodes sinensis*, *Ixodes persulcatus* and *Dermacentor silvarum* ticks. The transovarial transmission was seen in the *I*. *sinensis* ticks with a rate of 40%, but neither in *I*. *persulcatus* nor in *D*. *silvarum* ticks. *I*. *sinensis* ticks also have the ability to transmit SFTSV horizontally to uninfected mice at 7 days after feeding, but not for *I*. *persalcatus* or *D*. *silvarum* ticks. In the transstadial transmission of *I*. *persulcatus* and *D*. *silvarum* ticks, *I*. *persulcatus* ticks were tested negative from larvae to adults. But the *D*. *silvarum* ticks were tested positive from larvae to nymphs, with the positive rate of 100% (10/10) for engorged larval ticks and 81.25% (13/16) for molted nymphs. However, the mice bitten by SFTSV-infected *D*. *silvarum* nymphs were negative for SFTSV detection. Therefore, there is not enough evidence to prove the transstadial transmission of SFTSV in *I*. *persalcatus* and *D*. *silvarum* ticks.

## Author summary

Due to its wide distribution and high fatality rate (16%-30%), severe fever with thrombocytopenia syndrome (SFTS) has been listed in the top 10 priority diseases blueprint by the world health organization (WHO) in 2017. SFTSV is a novel phlebovirus in the *Bunyaviridae* family, and *Haemaphysalis longicornis* tick has been demonstrated as a competent vector of SFTSV by experimental transmission study and field study. However, there are many other tick species that infest human beings in the SFTS endemic regions. Therefore, it’s neccessary to query whether these tick species are capable of transmitting SFTSV. The authors found that in addition to *H*. *longicornis* ticks, *Ixodes sinensis* ticks also served as an efficient vector capable of transovarial transmitting SFTSV, therefore posing as a potential threat in causing the circulation of SFTSV. In contrast, *Dermacentor silvarum* and *Ixodes persulcatus* ticks might not serve as an efficient vector of transmitting SFTSV. This research will provide important reference for the surveillance of SFTSV and the disease prevention and control.

## Introduction

Severe fever with thrombocytopenia syndrome (SFTS) is an emerging infectious disease that was first recognized in 2009 in China[1], and subsequently reported in Korea[2, 3], Japan[4], and Vietnam[5]. The case fatality rate ranged from 16% to 30% that differed among various studies as there is no specific therapy available [6, 7, 8, 9, 10]. The casative virus, previously know as SFTS virus [SFTSV]), was recently renamed as Huaiyangshan banyangvirus, by the International Committee on Taxonomy of Viruses [11], as it belongs to the genus *Banyangvirus* in the family *Phenuiviridae*. *Haemaphysalis longicornis*, a tick species with a wide distribution and broad host range, has been demonstrated to be a competent vector of SFTSV, evidenced by sucessful SFTSV transmission through bites on mice in the experimental study[12, 13]. Epidemiological study also disclosed a strong association between *H*. *longicornis* tick presence and SFTS incidence, therefore corroborating the role of this tick specie in causing and transmitting human infection [14, 15, 16, 17].

On the other hand, SFTSV were also detected in several other tick species, including *Rhipicephalus microplus*, *Rhipicephalus sanguinensis*, and *Haemaphysalis concinna* ticks in SFTS endemic regions [18, 19]. In Korea, SFTSV has also been detected from *H*. *longicornis*, *Haemaphysalis flava*, *Ixodes nipponensis*, and *Amblyomma testudinarium* ticks in nature [3, 20]. All these findings indicated that these ticks might be involve in the circulation of SFTSV in the nature, suggesting the possibility of other tick vectors in haboring and transmitting SFTSV. However, the mere detection or isolation of SFTSV does not guarintine their capacity of acting as competent vectors. The competency of transmitting the virus and causing human infection was determined by complicated factors, including the capacity of amplifying and transmitting SFTSV, adequate infection rate of SFTSV in these tick species, and their adequate contact frequency with human beings. In the current study, we performed an epidemiological study to delineate the correlation between the abundance of predominant tick species and the human incidence of SFTS on county level in SFTS endemic region, and conducted the experimental transmission study to compare the different capacity of transmitting SFTSV among *Ixodes sinensis*, *Ixodes persulcatus*, and *Dermacentor silvarum* ticks.

## Materials and methods

### Determination of major tick species in the SFTS endemic regions

All laboratory-confirmed cases reported from 2010–2018 in China were retrieved from the Chinese Information System for Diseases Control and Prevention (CISDCP). Based on the data of the CISDCP, totally 25 provinces had reported the incidence of human SFTS cases. We analysed the distribution of the predominant tick species in China by checking literatures reporting the occurrence ticks species in China. Briefly, five main electronic databases (PubMed, ISI Web of Science, China WanFang database, China National Knowledge Infrastructure, and Chinese Scientific Journal Database) were searched for studies published between Jan, 1950 and May, 2017, using the following keywords: (“Tick” or “Ticks”) and “China” in all fields. We also checked the references in retrieved articles to reach more relevant articles. The information was collected using a standard form, including study date, study area at the county level, tick species identified, laboratory methods, and detection results for tick-borne pathogens. Based on literature review, *H*. *longicornis*, *R*. *microplus*, *D*. *silvarum*, *I*. *persulcatus* and *I*. *sinensis* ticks were determined to be the top 5 commonly seen tick species that bites human beings in China. The geographical distribution of the five tick species and the SFTS laboratory-confirmed cases from 2010 to 2018 in China are shown on the map (S1 Fig), which was created in ArcGIS 9.2 software (ESRI Inc., Redlands, CA, USA). The *R*. *microplus* ticks is the main ectoparasite of cattle and considered to be the most important external parasite impacting the cattle industry in the world. There are currently no reports of biting human beings by *R*. *microplus* ticks [21, 22]. Our laboratory had established colonies of *H*. *longicornis*, *I*. *sinensis*, *I*. *persulcatus* and *D*. *silvarum* ticks, which were used for the subsequent experimental transmission study. *H*. *longicornis* tick, which has been proved to be a competent vector of SFTSV, was evaluated in the current study as a positive control.

### SFTSV strain and culturing

The SFTSV strain WCH/97/HN/China/2011 used in this study was isolated from a human patient in Xinyang City, Henan province, China in 2011 [23], and maintained in Vero E6 Cell line with complete Dulbecco’s modified eagle’s medium (DMEM) with 10% fetal bovine serum, and 10U/mL penicillin and streptomycin. After the viral titer was determined by plaque assay, the virus was harvested for artificial infection of ticks by microinjection.

### Tick colony and experimental animals

*I*. *sinensis*, *I*. *persulcatus* and *D*. *silvarum* ticks were collected by flagging on vegetation in Zhejiang province, Heilongjiang Province, and Inner Mongolia Autonomous Region, China in 2017, respectively. We established SFTSV-free tick colonies in our laboratory from engorged female ticks. Briefly, the adults of *D*. *silvarum* ticks were allowed to feed on specific-pathogen free female New Zealand white rabbits weighting 2–3 kg, and the other ticks were allowed to feed on specific-pathogen free female BALB/c mice weighing 10–12 g. All the animals used in this study were supplied by the Laboratory Animal Center of Academy of Military Medical Sciences (Beijing, China). The fully engorged females were kept individually in an Intelligent Climate Cabinet (Saife Company, Ningbo City, China) with a relative humidity of 95 ± 5% at 26°C until they laid eggs. In each of tick species, 10 batches (30 eggs in each batch) of eggs were randomly sampled to screen for SFTSV by real-time reverse transcription PCR (rRT-PCR) assays as described [12], along with the corresponding engorged females. The eggs from the groups with both the mother tick and the filial eggs detected as SFTSV-negative were incubated to larvae. The larvae and the following nymphs were fed on BALB/c mice, and the molted adults were subjected to the trial.

### Artificial infection of ticks with SFTSV

For the artificial infection of adult ticks, the SFTSV-free colony of each tick species were infected with 1 μl SFTSV virus culture (3.65×10^6^ PFU/ml), or the same volume of phosphate buffered saline (PBS), by microinjection protocol through its anal pore as described [12]. The ticks which remained alive and active after injection were maintained till to lay eggs and further molt to larvae.

The artificial infection of larvae and nymphs was conducted through feeding on SFTSV-infected mice. Brief, two-week old mice were intraperitoneally inoculated with 200 μl of virus solution (3.65×10^6^ PFU/ml) or the same volume of PBS, and the infected mice were subsequently used to feed the ticks for 3–5 days until engorged [13].

### Transmission cycle of SFTSV in ticks

**Fig 1:**
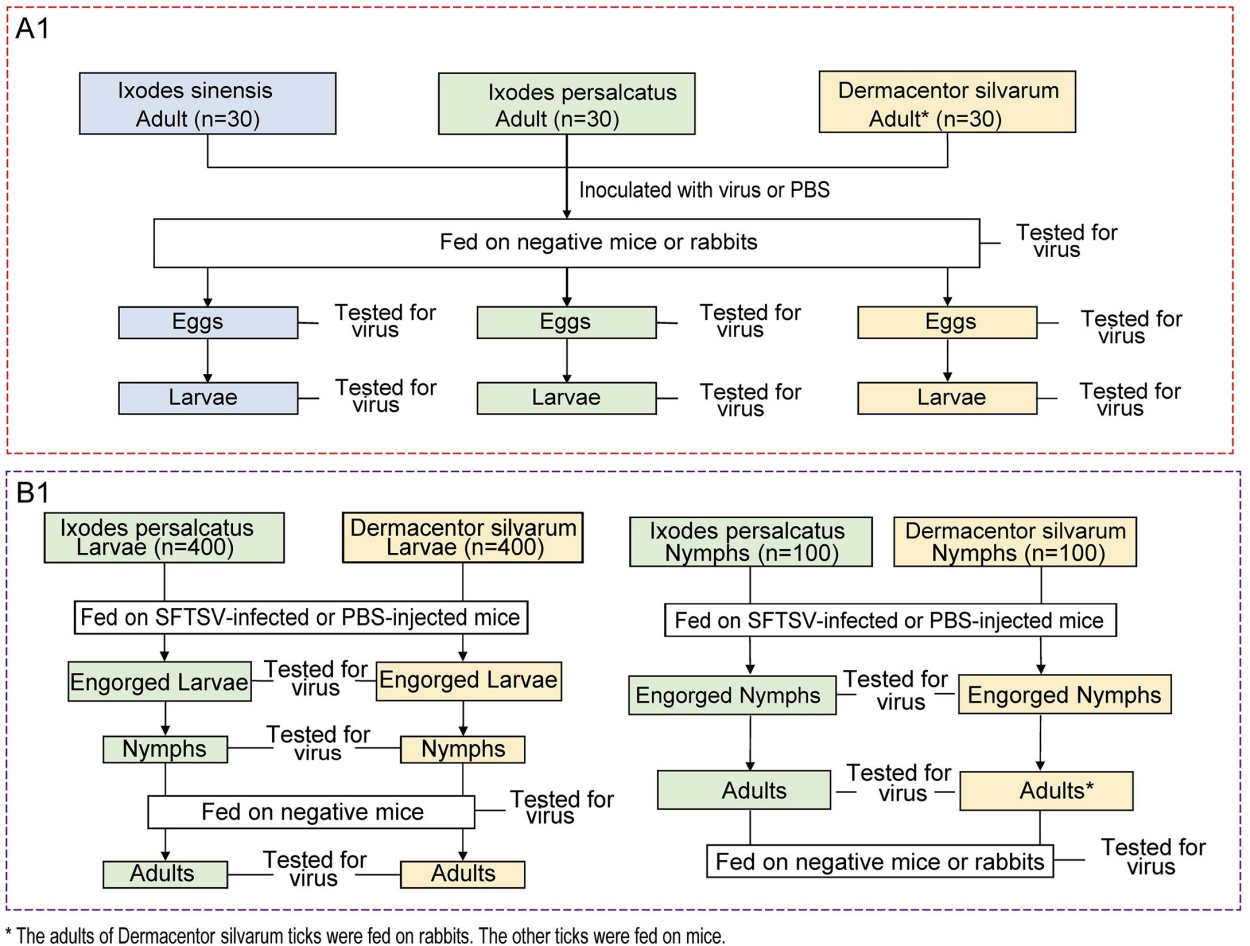
Framework of experimental transmission study of SFTSV. (A) Transovarial Transmission. Thirty adults for each three tick species that were microinjected with SFTSV cell culture dilution (3.65×10^6^ PFU/ml) were used as SFTSV group. Thirty adults for each three tick species microinjected with PBS were used as control group. (B) Transstadial Transmission. To determine transstadial transmission of SFTSV by two tick species, we fed larvae and nymphs on SFTSV-infected mice in the SFTSV group and fed larvae and nymphs on PBS-injected mice in the control group. In larval or nymph acquisition feeding, the engorged larvae or engorged nymphs were tested for SFTSV by RT-PCR before or after molting.

The transovarial transmission of SFTSV in *I*. *sinensis*, *I*. *persulcatus* and *D*. *silvarum* ticks and the transstadial transmission of SFTSV in *I*. *persulcatus* and *D*. *silvarum* ticks were conducted following the procedures (Fig 1). Thirty adults for each three tick species that were microinjected with SFTSV cell culture dilution, were used as SFTSV group, and thirty adults for each three tick species microinjected with PBS were used as control group. Two weeks after injection, the female ticks of *I*. *sinensis* and *I*. *persulcatus* ticks were fed on BALB/c mice, and the female ticks of *D*. *silvarum* ticks were fed on New Zealand white rabbits. The engorged female ticks were maintained until they laid eggs, which were allowed to hatch to larvae under the same conditions as described earlier. We screened subsequent larvae for SFTSV infection to assess the efficiency of transovarial transmission.

To determine transstadial transmission of SFTSV by two tick species, we fed larvae and nymphs on SFTSV-infected mice in the SFTSV group and fed larvae and nymphs on PBS-injected mice in the control group. In larval or nymph acquisition feeding, the engorged larvae or engorged nymphs were tested for SFTSV by rRT-PCR before or after molting. At each developmental stage, ticks were starved for 3 weeks between molting and the next feeding, each period of development of four tick species shown in S1 Table.

### Detection of SFTSV in ticks of different developmental stages

Ticks of different developmental stages were subjected to SFTSV detection by performing rRT-PCR. Briefly, total RNAs were extracted from egg pools (60/pool), larva pools (50/pool), engorged larva pools (10/pool), nymph pools (3/pool), engorged nymph pools (3/pool), and individual adult tick, and were then analyzed for SFTSV by rRT-PCR with specific primers and probes. The virus load was determined using rRT-PCR targeting the same gene segments as described [12]. The viral RNA was prepared as a positive control.

### Detection of SFTSV in animals

Serum samples were collected from the mice or rabbits 3 times (before tick feed, 1 week after tick engorgement, and 3 weeks after tick repletion). Total RNA was extracted from the sera by using a QIAamp Viral RNA mini kit, and subsequently used for detection of SFTSV by rRT-PCR as mentioned above. Using the viral antigen of the SFTSV strain WCH/97/HN/China/2011 from the Vero E6 cell line, we detected specific IgG against SFTSV by IFA (Indirect Immunofluorescence Assay), as described [12]. We measured antibody titers with serum dilution starting at 1:20. Samples from uninfected animals were taken at the same time for negative control use.

## Results

### Carrying days of SFTSV in adult ticks

Before the formal experiment, 60 female ticks from SFTSV-free colony of *H*. *longicornis*, *I*. *sinensis*, *I*. *persalcatu* and *D*. *silvarum* ticks by microinjection of SFTSV, five ticks were taken from each tick species to detect for SFTSV RNA on 0, 3, 6, 9, 12, 15, 18 and 21 days post injection. SFTSV genome was detected in the adult ticks for as long as 21 days in *H*. *longicornis* ticks, 18 days in *I*. *sinensis* ticks, 9 days in *I*. *persalcatus* ticks, and 6 days in *D*. *silvarum* ticks.

### Positive rate of SFTSV in adult ticks

For *I*. *sinensis*, *I*. *persalcatu*, *D*. *silvarum* and *H*. *longicornis* ticks, 60 female ticks of each tick species from an SFTSV-free colony were randomly and equally grouped into experimental and control group. Five days post injection, 6 live SFTSV-infected ticks for each tick species were screened for SFTSV infection. All (6/6) *I*. *sinensis* ticks, 66.7% (4/6) of *I*. *persalcatu* ticks, 33.3% (2/6) of *D*. *silvarum* ticks and 100% (6/6) of *H*. *longicornis* ticks were positive for detection of SFTSV RNA. Sequencing analysis based on the same gene segments as previously described[12] demonstrated that the virus derived from each of the infected tick species matched that of the inoculated strain. None of the negative control group had SFTSV detected (S2 Table).

### Transovarial transmission of SFTSV by ticks

Twelve live female ticks of *D*. *silvarum* ticks were fed on New Zealand white rabbits (4 ticks per rabbit), and twelve live female ticks of each of the other three tick species were fed on BALB/c mice (4 ticks per mouse) until the ticks detached from the animal. The engorged females were harvested and maintained to lay eggs (S3 Table). All the 15 pools of eggs laid by 5 infected *I*. *sinensis* ticks (3 pools from each tick) were SFTSV RNA positive. All 15 pools of eggs laid by 5 infected *I*. *persalcatus* ticks (3 pools from each tick) were SFTSV RNA negative. All 15 pools of eggs laid by 5 infected *D*. *silvarum* ticks (3 pools from each tick) were SFTSV RNA negative. None of the egg pools from the control group was positive (Table 1). Besides, all the 15 pools of eggs laid by 5 infected *H*. *longicornis* ticks (3 pools from each tick) were SFTSV RNA positive.

**Table 1:** Detection of SFTSV for four tick species in the transovarial transmission.

Group	Species	Project	Mother tick	Egg poll*	Larvae poll^†^
SFTSV Group	*I*. *sinensis*	No. tested	5	15	25
		% Positive ± SE	100	100	40 ± 14.1
	*I*. *persulcatus*	No. tested	5	15	25
		% Positive	0	0	0
	*D*. *silvarum*	No. tested	5	15	25
		% Positive	0	0	0
	*H*. *longicornis*	No. tested	5	15	25
		% Positive ± SE	100	100	80± 1.7
Control Group	*I*. *sinensis*	No. tested	5	15	25
		% Positive ± SE	0	0	0
	*I*. *persulcatus*	No. tested	5	15	25
		% Positive	0	0	0
	*D*. *silvarum*	No. tested	5	15	25
		% Positive	0	0	0
	*H*. *longicornis*	No. tested	5	15	25
		% Positive ± SE	0	0	0

*Eggs were tested in pools of 60.

†Larvae were tested in pools of 50.

When hatched to larvae, 10 of 25 pools derived from the infected *I*. *sinensis* ticks (5 pools from each tick) tested positive for SFTSV RNA. None of 25 pools derived from the infected *I*. *persalcatus* ticks (5 pools from each tick) tested positive for SFTSV RNA, none of 25 pools derived from the infected *D*. *silvarum* ticks (5 pools from each tick) tested positive for SFTSV RNA. For *H*. *longicornis* ticks, as a positive control, all of 25 pools larvae (5 pools from each tick) tested positive for SFTSV RNA. All larvae pools from four tick species of the control group tested negative (Table 1).

### Transstadial transmission of SFTSV by three tick species

As *I*. *persalcatus* and *D*. *silvarum* ticks were negative in hateched larvae, we further performed transstadial transmission of SFTSV by artificially infecting larvae. For *I*. *persalcatu*, *D*. *silvarum* and *H*. *longicornis* ticks, 400 larvae ticks negative for SFTSV of each tick species were artificially infected with SFTSV. In larval acquisition feeding, the engorged larvae were tested for SFTSV by rRT-PCR before and after molting (S4 Table). For *I*. *persulcatus* ticks, the engorged larval pools show a positive rate of 55% (11/20), and the molted nymphs was negative (0/20). For *D*. *silvarum* ticks, the positive rate was 100% (10/10) for engorged larval pools and 81.25% (13/16) for molted nymphs. For *H*. *longicornis* ticks, the positive rate was 100% (20/20) for engorged larval pools and 100% (20/20) for molted nymphs. None from the control group of each tick species were positive for SFTSV RNA (Table 2). These results indicated that transstadial transmission of SFTSV from larvae to nymphs occurred for *H*. *longicornis* and *D*. *silvarum* ticks, but not for *I*. *persulcatus* ticks. The *I*. *sinensis* ticks were not tested for the transstadial transmission from larvae to nymphs due to inadequate tick number.

**Table 2:** Detection of SFTSV for three tick species in the transstadial transmission.

Group	Species	Project	Larval molted to Nymph		Nymph molted to Adult	
			Engorged larvae pool^‡^	Nymph poll^§^	Engorged nymph poll^#^	Adult^¶^
SFTSV Group	*I*. *persulcatus*	No. tested	20	20	10	20
		% Positive	55	0	100	0
	*D*. *silvarum*	No. tested	10	16	10	16
		% Positive	100	81.25	100	0
	*H*. *longicornis*	No. tested	20	20	10	20
		% Positive	100	100	100	50
Control Group	*I*. *persulcatus*	No. tested	20	20	10	20
		% Positive	0	0	0	0
	*D*. *silvarum*	No. tested	10	16	10	16
		% Positive	0	0	0	0
	*H*. *longicornis*	No. tested	20	20	10	20
		% Positive	0	0	0	0

^‡^Engorged larvae were tested in pools of 10.

^§^Nymphs were tested in pools of 3.

^#^Engorged nymphs were tested in pools of 3.

^¶^Adult was tested on pools of 1

For the transstadial transmission study from nymph to adult, 100 negative nymphs of *I*. *persalcatus*, *D*. *silvarum* and *H*. *longicornis* ticks were artificially infected with SFTSV. Similarly, the engorged larvae were tested for SFTSV by rRT-PCR before and after molting (S4 Table). For *I*. *persalcatus* and *D*. *silvarum* ticks, 10 pools of engorged nymphs of each tick species were all found to be positive for SFTSV and none of the adult ticks of each tick species derived from the nymphs were infected with SFTSV. For *H*. *longicornis* ticks, the positive rate was 100% (10/10) for engorged nymphs pools and 50% (10/20) for molted adult ticks. Control group of three tick species were also tested negative (Table 2). These results indicated no transstadial transmission of SFTSV from nymphs to adult ticks for either *I*. *persulcatus* or *D*. *silvarum* ticks. The *I*. *sinensis* ticks were not tested for the transstadial transmission from nymph to adult due to inadequate tick number.

### Transmission of SFTSV to animals by ticks

**Fig 2:**
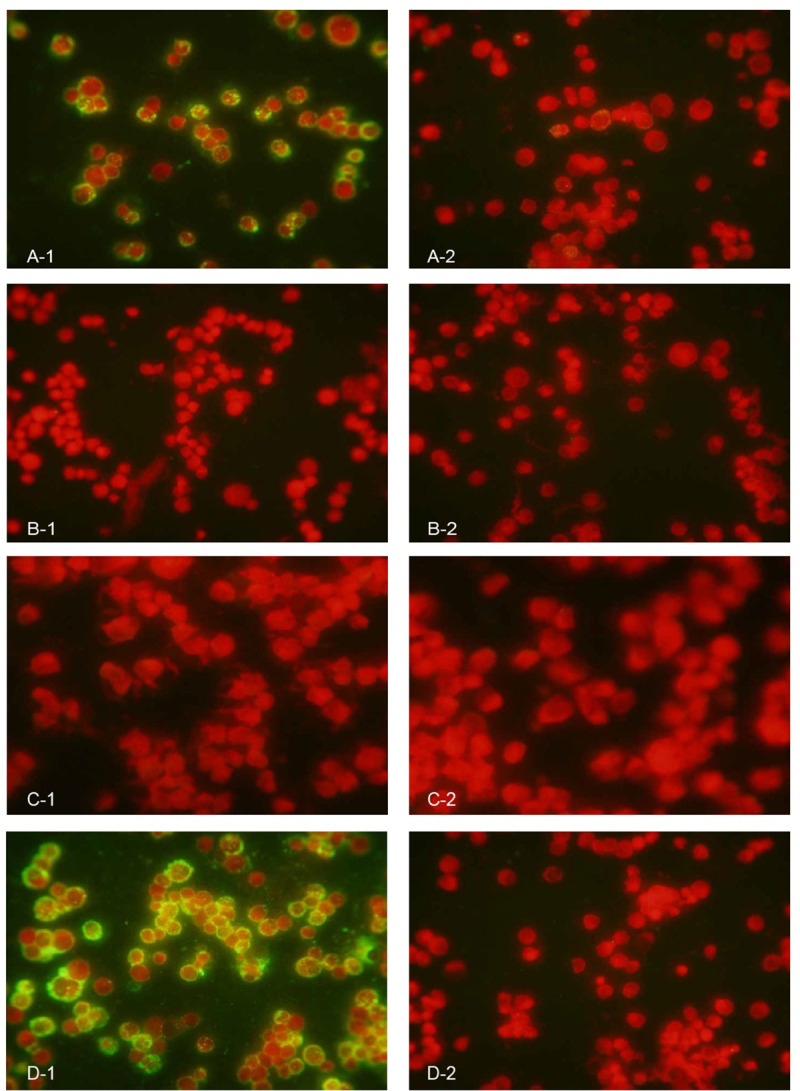
Detection of severe fever with thrombocytopenia syndrome virus (SFTSV) antibodies in serum samples from animals fed by SFTSV-infected ticks through immunofluorescence assay. A) Serum samples from the mouse bitten by I. sinensis ticks reacting with SFTSV-infected Vero E6 cells (A-1 SFTSV group, A-2 Control group). B) Serum samples from the mouse bitten by I. persulcatus ticks reacting with SFTSV-infected Vero E6 cells (B-1 SFTSV group, B-2 Control group). C) Serum samples from the rabbit bitten by D. silvarum ticks reacting with SFTSV-infected Vero E6 cells (C-1 SFTSV group, C-2 Control group). D) Serum samples from the rabbit bitten by H. longicornis ticks reacting with SFTSV-infected Vero E6 cells (D-1 SFTSV group, D-2 Control group).

During the transovarial transmission experiment, a total of 18 BALB/c mice and 6 New Zealand white rabbits were used for feeding ticks. For the injected adult tick feeding, 1 of 3 BALB/c mice fed by the SFTSV-infected adult *I*. *sinensis* ticks was positive for detection of SFTSV RNA 7 days after the ticks detached. All the 3 mice bitten by the SFTSV-infected adult *I*. *persulcatus* ticks and the 3 rabbits bitten by the SFTSV-infected adult *D*. *silvarum* ticks were negative. As a positive control, 3 of 3 BALB/c mice fed by the SFTSV-infected adult *H*. *longicornis* ticks was positive for SFTSV RNA detection 7 days after the ticks detachment. The animals bitten by ticks from the control group were also negative. We used IFA to test serum samples from the mice collected before and 3 weeks after detachment of ticks at different developing stages; the animals having positive result for SFTSV RNA detection showed seroconversion against SFTSV (Table 3, Fig 2)

**Table 3:** Animals used in the transovarial transmission cycle of SFTSV for four tick species.

Group	Project	*I*. *sinensis*	*I*. *persulcatus*	*D*. silvarum*	*H*. *longicornis*
SFTSV group	No. mice or rabbits for feed and detection	3	3	3	3
	No. ticks fed on each mouse or rabbit	4	4	4	4
	No. positive by RT-PCR**/**total	1/3	0/3	0/3	3/3
	No. positive by IFA**/**total	1/3	0/3	0/3	3/3
	IFA antibody titer	1:80	-	-	1:1280
Control group	No. mice or rabbits for feed and detection	3	3	3	3
	No. ticks fed on each mouse or rabbit	4	4	4	4
	No. positive by RT-PCR**/**total	0/3	0/3	0/3	0/3
	No. positive by IFA**/**total	0/3	0/3	0/3	0/3
	IFA antibody titer	-	-	-	-

*The adults of D. silvarum ticks were fed on New Zealand white rabbits.

During the transstadial transmission experiment, a total of 16 BALB/c mice and 2 New Zealand white rabbits were used for feeding ticks. For the nymph ticks feeding. The 2 mice bitten by the positive nymphs of *D*. *silvarum* ticks were negative. None of 2 mice bitten by the nymphs of *I*. *persulcatus* ticks were positive. As a positive control, 2 of 2 BALB/c mice fed by the nymphs of *H*. *longicornis* ticks was positive. For the adult ticks feeding, the animals bitten by the adults of *D*. *silvarum* and *I*. *persulcatus* ticks were negative. The animals bitten by the adults of *H*. *longicornis* ticks was positive. The animals bitten by ticks from the control group were negative for SFTSV detection. The animals having positive result for SFTSV RNA detection showed seroconversion against SFTSV (Table 4).

**Table 4:** Animals used in the transstadial transmission cycle of SFTSV for three tick species.

T Group	Project	Nymph molted by infected larval			Adult molted by infected nymph		
		*I*. *persulcatus*	*D*. *silvarum*	*H*. *longicornis*	*I*. *persulcatus*	*D*. silvarum*	*H*. *longicornis*
SFTSV group	No. mice or rabbits for feed and detection	2	2	2	1	1	1
	No. ticks fed on each mouse or rabbit	10	10	10	5	5	5
	No. positive by RT-PCR**/**total	0/2	0/2	2/2	0/1	0/1	1/1
	No. positive by IFA**/**total	0/2	0/2	2/2	0/1	0/1	1/1
	IFA antibody titer	-	-	1:640	-	-	1:1280
Control group	No. mice or rabbits for feed and detection	2	2	2	1	1	1
	No. ticks fed on each mouse or rabbit	10	10	10	5	5	5
	No. positive by RT-PCR**/**total	0/2	0/2	0/2	0/1	0/1	0/1
	No. positive by IFA**/**total	0/2	0/2	0/2	0/1	0/1	0/1
	IFA antibody titer	-	-	-	-	-	-

*The adults of *D*. *silvarum* ticks were fed on New Zealand white rabbits.

## Discussion

The current study supported that in addition to *H*. *longicornis* ticks, *I*. *sinensis* ticks also served as an efficient vector capable of transovarial transmitting SFTSV, therefore posing as a potential threat in causing the circulation of SFTSV. In contrast, *D*. *silvarum* and *I*. *persalcatus* ticks might not serve as an efficient vector of transmitting SFTSV.

Experimental transstadial transmission of *D*. *silvarum* ticks for SFTSV RNA was positive in the larvae to nymph routes, but neither in the nymph to adult route nor in transovarial transmission. The mice bitten by SFTSV-positive nymphs of *D*. *silvarum* ticks were negative. There is not enough evidence to prove the transstadial transmission of SFTSV in *D*. *silvarum* ticks. We speculate that the possible reason is the time from larval to nymph molting stage of *D*. *silvarum* ticks was short. SFTSV in nymph of *D*. *silvarum* ticks was only the residue of engorged larvae, but no SFTSV proliferation in nymph of *D*. *silvarum* ticks. Further studies are needed to confirm the transstadial transmission of SFTSV in *D*. *silvarum* ticks. This presented as the first experimental studies on evaluating other tick species than *H*. *longicornis* ticks to serve as vectors of the SFTSV.

Since the discovery of SFTSV, much interest has been put to identify the potential transmission vectors of the pathogen, but mostly via the epidemiological study to perform the molecular test of SFTSV RNA in ticks. A previous study screening *H*. *longicornis* ticks showed that SFTSV was detected in 4.93% of the ticks in Hubei and Henan provinces in central China[18] and 2.2% of the ticks in Shandong province[24] in eastern China. Except for *H*. *longicornis*, SFTSV was detected in 0.61% of *R*. *microplus* ticks in central China[18]. In addition, a recent investigation in Xinjiang Uygur Autonomous Region found the infection with SFTSV in *D*. *nuttalli* and *Hyalomma asiaticum* ticks [25]. Besides China, SFTSV was detected in 4.77% of *H*. *longicornis* ticks, 1.15% of *H*. *flava* ticks, and 20% in *A*. *testudinarium* ticks in South Korea [20]. SFTSV was also detected in *I*. *nipponensis* ticks in South Korea [3]. A study in Japan suggested that SFTSV positivity rates were considered to be very low in ticks and viral loads were also very limited, as SFTSV was not detected in 2222 adult and nymph ticks collected from vegetation [26]. These results suggested that several tick species might act as vectors for SFTSV, however, the presence of viral RNA in ticks does not verify that the tick can transmit the virus. Here in addition to *H*. *longicornis* ticks, we have tested the most commonly seen tick species in SFTS endemic regions for their role in transmitting SFTSV.

Here a differential capacity of transmitting SFTSV was displayed. Among the tested ticks, *I*. *sinensis* was shown to be the only competent one, with transovarial transmission competency of SFTSV that was next only to *H*. *longicornis* ticks. SFTSV RNA was detected in eggs and larvae of *I*. *sinensis* ticks. And one mouse fed by the SFTSV-infected adult *I*. *sinensis* ticks became infected, evidenced by both detection of SFTSV RNA and seroconversion. However, the transstadial transmission from larval to nymph and from nymph to adult was not accomplished, due to the low quality of colonies that were kept in the laboratory. Still, the current available results provided adequate evidence that the *I*. *sinensis* ticks played roles in maintaining and transmitting SFTSV among natural environment. According to the epidemiological study, *I*. *sinensis* tick was predominantly distributed in the middle China, as well as Anhui, Zhejiang, and Fujian provinces, where both *H*. *longicornis* and *I*. *sinensis* ticks were abundant during the SFTS epidemic season, both could act as reservoirs and vectors for the transmission of SFTSV in the nature. Until recently, there has been no report on the detection of SFTSV in *I*. *sinensis* ticks, probably due to its low density in local endemic region, thus more efforts should be put on this tick species, in order to enhance the understanding on this tick species.

In the previous paper[12], we have shown that SFTSV was disseminated in ovaries and salivary glands, indicating the infected *H*. *longicornis* ticks could transovarially and transstadially transmit SFTSV successfully. In this study, in addition SFTSV could be transmitted by *H*. *longicornis* ticks in both transovarial and transstadial way. Moreover, the maintenance of SFTSV genetic sequences in the adult *H*. *longicornis* ticks were determined to last as long as 21 days, significantly longer than those of other three tick species (18 days in *I*. *sinensis* ticks, 9 days in *I*. *persalcatus* ticks, and 6 days in *D*. *silvarum* ticks). Long carrying days of SFTSV in adults of *H*. *longicornis* ticks laid the foundation of transmission of SFTSV.

In addition to *H*. *longicornis* ticks, we demonstrated that *I*. *sinensis* ticks also served as an efficient vector capable of transstadial transmitting SFTSV by experimental study. Less important role of SFTSV transmission was implicated for *D*. *silvarum* and *I*. *persalcatus* ticks. Further study of tick-host-pathogen interactions are needed to explore the effect of abiotic factors on SFTSV transmission during the animal experiment.

## Supporting information

S1 Fig Geographical distribution of five tick species and SFTS laboratory-confirmed cases from 2010–2018 in China. The background is the SFTS laboratory-confirmed cases from 2010 to 2018 in China. A) Geographical distribution of *H*. *longicornis* ticks. B) Geographical distribution of *R*. *microplus* ticks. C) Geographical distribution of *D*. *silvarum* ticks. D) Geographical distribution of *I*. *persulcatus* ticks. E) Geographical distribution of *I*. *sinensis* ticks.
(TIF)

S1 Table The mean (± standard error) days of each development stage for four tick species. (DOCX)

S2 Table Number of artificially infected ticks in the study. (DOCX)

S3 Table Ticks and egg number (± standad error) used in the transovarial transmission. (DOCX)

S4 Table Ticks number used in the transstadial transmission of SFTSV for three tick species. (DOCX)
