# Supplementary figures and images for: Role of three tick species in the maintenance and transmission of Severe Fever with Thrombocytopenia Syndrome Virus
Source: PLoS Negl Trop Dis. 2020 Jun 10;14(6):e0008368. doi: 10.1371/journal.pntd.0008368 (PMC7307786; doi:10.1371/journal.pntd.0008368)

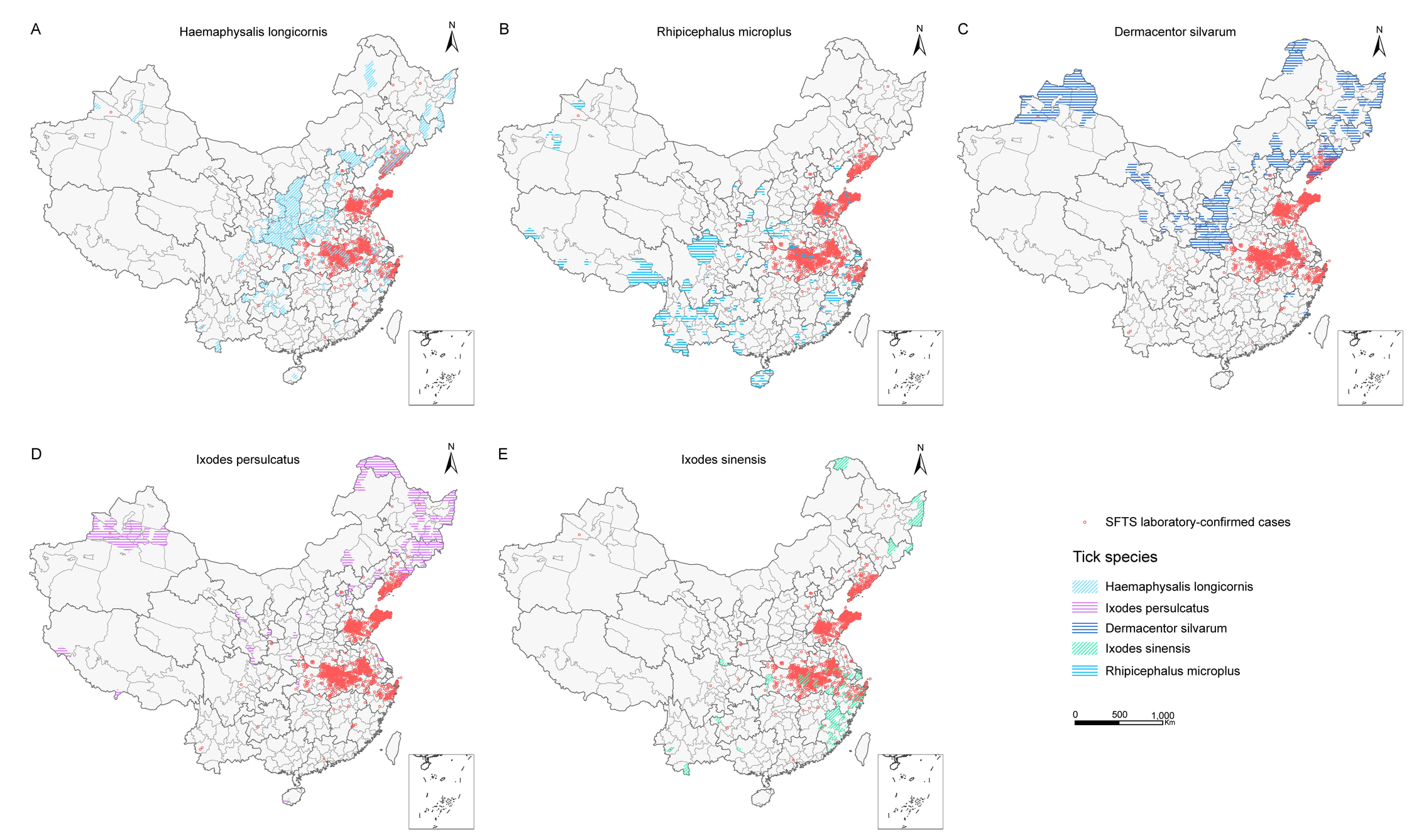

Supplement: S1 Fig — The background is the SFTS laboratory-confirmed cases from 2010 to 2018 in China. A) Geographical distribution of H. longicornis ticks. B) Geographical distribution of R. microplus ticks. C) Geographical distribution of D. silvarum ticks. D) Geographical distribution of I. persulcatus ticks. E) Geographical distribution of I. sinensis ticks. (TIF) [file pntd.0008368.s001.tif]
