# Supplementary material for: Role of three tick species in the maintenance and transmission of Severe Fever with Thrombocytopenia Syndrome Virus
Source: PLoS Negl Trop Dis. 2020 Jun 10;14(6):e0008368. doi: 10.1371/journal.pntd.0008368 (PMC7307786; doi:10.1371/journal.pntd.0008368)
Supplement: S1 Table — (DOCX) [file pntd.0008368.s002.docx]

| Period | *I. sinensis* | |  | *I. persulcatus* | |  | *D. silvarum* | |  | *H. longicornis* | |
| --- | --- | --- | --- | --- | --- | --- | --- | --- | --- | --- | --- |
|  | SFTSV group | Control group |  | SFTSV group | Control group |  | SFTSV group | Control group |  | SFTSV group | Control group |
| Adult feeding period | 12.8 ± 4.1 | 11.9 ± 3.4 |  | 5.41 ± 0.90 | 4.67 ± 0.58 |  | 12.50± 0.76 | 13.00 ± 0.58 |  | 9.12 ± 1.42 | 9.04 ± 1.84 |
| Preovipositon period | 22.52 ± 3.71 | 25.33 ± 2.08 |  | 5.67 ± 1.49 | 5.33 ± 0.58 |  | 5.88 ± 0.83 | 7.00 ± 0.58 |  | 8.20 ± 2.39 | 7.8 ± 1.80 |
| Oviposition period | 10.21 ± 3.23 | 11.33 ± 1.52 |  | 8.75 ± 1.21 | 8.67 ± 0.58 |  | 21.63 ± 1.19 | 22.00 ± 1.15 |  | 7.73 ± 1.69 | 7.7 ± 1.72 |
| Egg hatching period | 8.50 ± 1.52 | 8.33 ± 1.53 |  | 16.17 ± 2.66 | 17.33 ± 2.31 |  | 9.63 ± 1.50 | 9.00 ± 1.00 |  | 38.5 ± 1.24 | 38.4 ± 1.46 |
| Larva feeding period | N/A | N/A |  | 3.42 ± 0.80 | 3.00 ± 1.00 |  | 4.13 ± 0.64 | 4.00 ± 0.58 |  | 3.52 ± 0.64 | 3.4± 0.63 |
| Larva molting period | N/A | N/A |  | 18.17 ± 1.75 | 18.67 ± 2.52 |  | 6.38 ± 1.06 | 7.00 ± 1.53 |  | 22.20 ± 1.67 | 21.7 ± 1.64 |
| Nymph feeding period | N/A | N/A |  | 4.25 ± 0.62 | 4.67 ± 0.58 |  | 6.00 ± 0.93 | 5.00 ± 1.00 |  | 5.8 ± 1.28 | 5.55 ± 1.20 |
| Nymph molt period | N/A | N/A |  | 80.17 ± 4.04 | 79.33 ± 3.06 |  | 15.25 ± 1.28 | 15.00 ± 1.00 |  | 27.15± 1.35 | 26.70 ± 1.42 |

**S1 Table.** The mean (± standard error) days of each development stage for four tick species
