# Supplementary material for: Role of three tick species in the maintenance and transmission of Severe Fever with Thrombocytopenia Syndrome Virus
Source: PLoS Negl Trop Dis. 2020 Jun 10;14(6):e0008368. doi: 10.1371/journal.pntd.0008368 (PMC7307786; doi:10.1371/journal.pntd.0008368)
Supplement: S2 Table — (DOCX) [file pntd.0008368.s003.docx]

|  | *I. sinensis* | |  | *D. silvarum^*^* | |  | *I. persulcatus* | |  | *H. longicornis* | |
| --- | --- | --- | --- | --- | --- | --- | --- | --- | --- | --- | --- |
| No*.* | SFTSV | Control |  | SFTSV | Control |  | SFTSV | Control |  | SFTSV | Control |
| Total injected ticks | 30 | 30 |  | 30 | 30 |  | 30 | 30 |  | 30 | 30 |
| Dead ticks | 12 | 12 |  | 12 | 12 |  | 12 | 12 |  | 12 | 12 |
| Ticks used for RT-PCR detection (positive/all) | 6 (6/6) | 6 (0/6) |  | 6 (2/6) | 6 (0/6) |  | 6 (4/6) | 6 (0/6) |  | 6 (6/6) | 6 (0/6) |
| Ticks fed on mice or rabbit (4/mice or rabbit) | 12 | 12 |  | 12 | 12 |  | 12 | 12 |  | 12 | 12 |
| Engorged ticks | 5 | 5 |  | 5 | 5 |  | 5 | 5 |  | 5 | 5 |
| ^*^ The adults of D. silvarum ticks were fed on New Zealand white rabbits. | | | | | | | | | | | |

S2 Table. Number of artificially infected ticks in the study
