# Supplementary material for: Role of three tick species in the maintenance and transmission of Severe Fever with Thrombocytopenia Syndrome Virus
Source: PLoS Negl Trop Dis. 2020 Jun 10;14(6):e0008368. doi: 10.1371/journal.pntd.0008368 (PMC7307786; doi:10.1371/journal.pntd.0008368)
Supplement: S3 Table — (DOCX) [file pntd.0008368.s004.docx]

| Source | *I. sinensis* | |  | *I. persulcatus* | |  | *D. silvarum* | |  | *H. longicornis* | |
| --- | --- | --- | --- | --- | --- | --- | --- | --- | --- | --- | --- |
|  | SFTSV | Control |  | SFTSV | Control |  | SFTSV | Control |  | SFTSV | Control |
| Eggs | 1160.4 ± 66.7 | 1188.2 ± 45.7 |  | 1216.4 ± 77.8 | 1337.4 ± 94.6 |  | 1558.2 ± 63.8 | 1610.8 ± 17.0 |  | 1350.6 ± 47.7 | 1436.6 ± 159.9 |
| Egg for detection of each tick (eggs × pools） | 60×3 | 60×3 |  | 60×3 | 60×3 |  | 60×3 | 60×3 |  | 60×3 | 60×3 |
| Eggs left | 980.4 ± 66.7 | 1008.2 ± 45.7 |  | 1036.4 ± 77.8 | 1157.4 ± 94.6 |  | 1378.2 ± 63.8 | 1430.8 ± 17.0 |  | 1125.6 ± 47.7 | 1256.6 ± 159.9 |
| Hatched larvae | 832.6 ± 56.2 | 799.6 ± 71.6 |  | 818.0 ± 45.9 | 799.0 ± 54.6 |  | 1170.6 ± 37.5 | 1247.4 ± 31.7 |  | 889 ± 66.2 | 847.4 ± 114.2 |
| Larval for detection (larval × pools) | 50×5 | 50×5 |  | 50×5 | 50×5 |  | 50×5 | 50×5 |  | 50×5 | 50×5 |

S3 Table. Ticks and egg number (± standad error) used in the transovarial transmission
