# Supplementary material for: Role of three tick species in the maintenance and transmission of Severe Fever with Thrombocytopenia Syndrome Virus
Source: PLoS Negl Trop Dis. 2020 Jun 10;14(6):e0008368. doi: 10.1371/journal.pntd.0008368 (PMC7307786; doi:10.1371/journal.pntd.0008368)
Supplement: S4 Table — (DOCX) [file pntd.0008368.s005.docx]

|  |  | *I. persulcatus* | |  | *D. silvarum* | |  | *H. longicornis* | |
| --- | --- | --- | --- | --- | --- | --- | --- | --- | --- |
| Period | No. | SFTSV group | Control group |  | SFTSV group | Control group |  | SFTSV group | Control group |
| Larval molted to Nymph | Larvae for artificially infected | 400 | 400 |  | 400 | 400 |  | 400 | 400 |
|  | Mice for artificially infected | 5 | 5 |  | 5 | 5 |  | 5 | 5 |
|  | Larvae fed on each mouse | 80 | 80 |  | 80 | 80 |  | 80 | 80 |
|  | Engorged larvae | 301 | 315 |  | 267 | 286 |  | 325 | 318 |
|  | Larvae left | 101 | 115 |  | 167 | 186 |  | 125 | 118 |
|  | Nymphs | 86 | 92 |  | 74 | 82 |  | 96 | 89 |
| Nymph molted to Adult | Nymphs for artificially infected | 100 | 100 |  | 100 | 100 |  | 100 | 100 |
|  | Mice for artificially infected | 5 | 5 |  | 5 | 5 |  | 5 | 5 |
|  | Nymphs fed on each mouse | 20 | 20 |  | 20 | 20 |  | 20 | 20 |
|  | Engorged nymphs | 79 | 81 |  | 74 | 69 |  | 85 | 76 |
|  | Engorged nymphs left | 49 | 51 |  | 44 | 39 |  | 55 | 46 |
|  | Adult | 27 | 29 |  | 25 | 24 |  | 31 | 28 |

**S4 Table.** Ticks number used in the transstadial transmission of SFTSV for three tick species
